# Can TPO as Photoinitiator Replace “Golden Mean” Camphorquinone and Tertiary Amines in Dental Composites? Testing Experimental Composites Containing Different Concentration of Diphenyl(2,4,6-trimethylbenzoyl)phosphine Oxide

**DOI:** 10.3390/ijms231911594

**Published:** 2022-09-30

**Authors:** Andrea Kowalska, Jerzy Sokołowski, Małgorzata Iwona Szynkowska-Jóźwik, Tomasz Gozdek, Karolina Kopacz, Kinga Bociong

**Affiliations:** 1Department of General Dentistry, Medical University of Lodz, 92-213 Lodz, Poland; 2Faculty of Chemistry, Institute of General and Ecological Chemistry, Lodz University of Technology, Zeromskiego 116, 90-543 Lodz, Poland; 3Institute of Polymer & Dye Technology, Lodz University of Technology, Stefanowskiego 12/16, 90-924 Lodz, Poland; 4“DynamoLab” Academic Laboratory of Movement and Human Physical Performance, Medical University of Lodz, Pomorska 251, 92-215 Lodz, Poland; 5Department of Health Sciences, Medical University of Mazovia, Rydygiera 8, 01-793 Warszawa, Poland

**Keywords:** photoinitiator, TPO, camphorquinone, dental composite, experimental composite, resin-based composite, restorative dentistry, preventive dentistry

## Abstract

The aim of this research was to compare the biomechanical properties of experimental composites containing a classic photoinitiating system (camphorquinone and 2-(dimethylami-no)ethyl methacrylate) or diphenyl(2,4,6-trimethylbenzoyl)phosphine oxide (TPO) as a photoinitiator. The produced light-cured composites consisted of an organic matrix-Bis-GMA (60 wt.%), TEGDMA (40 wt.%) and silanized silica filler (45 wt.%). Composites contained 0.27; 0.5; 0.75 or 1 wt.% TPO. Vickers hardness, microhardness (in the nanoindentation test), diametral tensile strength, resistance to three-point bending and the CIE L* a* b* colorimetric analysis was performed with each composite produced. The highest average Vickers hardness values were obtained for the composite containing 1 wt.% TPO (43.18 ± 1.7HV). The diametral tensile strength remains on regardless of the type and amount of photoinitiator statistically the same level, except for the composite containing 0.5 wt.% TPO for which DTS = 22.70 ± 4.7 MPa and is the lowest recorded value. The highest average diametral tensile strength was obtained for the composite containing 0.75 wt.% TPO (29.73 ± 4.8 MPa). The highest modulus of elasticity characterized the composite containing 0.75 wt.% TPO (5383.33 ± 1067.1 MPa). Composite containing 0.75 wt.% TPO has optimal results in terms of Vickers hardness, diametral tensile strength, flexural strength and modulus of elasticity. Moreover, these results are better than the parameters characterizing the composite containing the CQ/DMAEMA system. In terms of an aesthetic composite containing 0.75 wt.%. TPO is less yellow in color than the composite containing CQ/DMAEMA. This conclusion was objectively confirmed by test CIE L* a* b*.

## 1. Introduction

In every branch of medicine doctors use golden means to treat their patients, but is it always the best solution for every situation? In operative dentistry, especially dental materials, dentists use most often composites to fill most of the dental cavities. Do dentists focus on ingredients of composite they use? Is it possible that 1% of a whole composite can change the aesthetic and quality of dental composite? Dentists accepted the advantages and disadvantages of golden mean which is camphorquinone. The technology and science are continuous and rapid processes, which evolve every year. Perhaps modern dentists will not have to compromise between quality and aesthetics aspects.

Camphoroquinone (CQ) is well-known photoinitiator used in most dental composites since 1975 [1]. It is alpha-diketone in a form of yellow powder, the range of absorbance is 360–510 nm [2] and the maximum of absorbance is 468 nm [2,3]. To speed up the process of polymerization and also deep of cure the tertiary amines are used as a co-initiators [1,4,5,6]. The molecules of tertiary amines are producing the yellowing effect on long-term composite filling due to their reactivity and oxidation [5]. The CQ is yellow powder, which has poor bleaching properties [7]. It has many advantages: First, most dental curing units have the range of light suitable for this photoinitiator, and second, it tested well in terms of the effect on strength, hardness, deep of cure, water sorption, polymerization shrinkage of composites that contain it. Unfortunately, it has some disadvantages as well. One of them is yellow color, which causes color-matching problems, especially when lighter shades are desired [5,8,9]. This color is caused by unreacted CQ. Another disadvantage is the cytotoxic effect of unconsumed CQ to pulp cells, which means that this photoinitiator is not as biocompatible as it was assumed to be [10,11,12]. CQ requires a reducing agent to initiate the polymerization effect, so CQ alone is not as efficient as CQ incorporated with tertiary amines [13]. However, tertiary amines can produce undesirable quaternary ammonium salts, which reduces the longevity of adhesion to dentin upon aging of composite [14,15].

The photoinitiator worth attention is diphenyl(2,4,6-trimethylbenzoyl)phosphine oxide (TPO). TPO is type-1 photoinitiator, which does not require additional initiators such as tertiary amines [9,14,16]. Eliminating co-initiators increased the stability of color upon aging [16,17]. This photoinitator has narrow wavelength absorption range: 380–425 nm and the maximum is 400 nm [17]. TPO requires special dental curing unit–new type which range of light is not only adapted to CQ range but it should be wider, like multipeak light curing system [18,19,20]. This photoinitiator is more effective than CQ because it delivers two free radicals during alpha cleavage, not one like CQ does [19]. However, TPO has also some disadvantages. First, TPO-based composites generate higher polymerization stresses than CQ-controls. The second disadvantage is the lower depth of cure of composites including TPO compared with CQ-containing mixture [20]. These photo-initiators are less suitable for water-containing adhesive systems due to the hydrophobic three-phenyl groups [21,22]. According to Van Der Lann et al., TPO has no toxic effect on pulp [2].

Most articles referring to modified photoinitiator systems have investigated one concentration of TPO and compared it with CQ and different tertiary amines [19,21,23,24]; however, our research compares four different concentrations according to the golden mean, which gives us opportunity to evaluate influence of TPO amount on composite properties. The testing hypothesis is to compare the properties of experimental composites containing different concentration of TPO to properties of CQ and tertiary amines in experimental dental composite. Additionally, the aim of this article was to assess the influence of concentration of TPO on microhardness, flexural strength, diameter tensile strength and color. The null hypothesis is that dental resin containing TPO performs no worse properties than composites with CQ/tertiary amines.

## 2. Materials and Methods

The compositions of the experimental composites that were tested in this study are shown in Table 1. The control group was dental resin containing CQ/tertiary amine because it is the most common photoinitiator system in dental resin composites. As a co-initiator to CQ, 2-(Dimethylamino)ethyl methacrylate (DMAEMA) (Sigma-Aldrich Inc., St. Louis, MO, USA) was used. The matrix of experimental composites was composed of 60 wt.%. bisphenol A glycerolate dimethacrylate (Bis-GMA) (Sigma-Aldrich Inc., St. Louis, MO, USA), 40 wt.% triethylene glycol dimethacrylate (TEGDMA) (Sigma-Aldrich Inc., St. Louis, MO, USA) and 0.1 wt.% 2,6-Di-tert-butyl-4-methylphenol (BHT) (Sigma-Aldrich Inc., St. Louis, MO, USA) as an inhibitor of polymerization. The composite was filled with 45 wt.% of silica (Arsil, Zakłady Chemiczne “RUDNIKI” S.A., Rudniki, Poland). Sillica, before use, was silanized with γ-methacryloxypropyltrimethoxy silane (Unisil Sp. z o. o., Tarnów, Poland). The composites were manually mixed (with an agate mortar, the laboratory method of producing composites) till a smooth paste was achieved at room temperature without daylight and artificial light. All samples were cured with polywave Valo Lamp Ultradent Products Inc., South Jordan, UT, USA) with three irradiance outputs (1000 mW/cm^2^, 1450 mW/cm^2^ and 3200 mW/cm^2^) and a light range of 395–510 nm. After multiple tries, the optimal curing duration was 20 s per 2 mm of material high. After this, the samples had optimal features.

The samples that were used to hardness and diametral tensile strength testing were cylindrical (3 mm high and 6 mm diameter). The material was put into silicon molds and then irradiated on both sides. The Vickers hardness (HV) test was performed as a first examination. Eleven samples were tested of every composite. It was determined with semiautomatic hardness tester (ZHV2-m Zwick/Röell, Ulm, Germany). The diamond shape as a square-based pyramid with apex angle 136 degree is used and the indenter was loaded with 9.81 N. The contact of pyramid and sample was 10 s.

The microhardness of the composites was tested with the NanoTest 600 (Micromaterials Ltd., Wrexham, UK) using a Berkovich indenter. The maximum force was 10 mN, the loading and unloading speed was dP/dt = 0.5 mN/s. The measurements were carried out in controlled conditions of temperature (T = 20 °C) and relative humidity (60 ± 5%). The samples that were tested was 2 mm high and 10 mm in diameter. The composite microhardness and reduced modulus were calculated on the basis of the unloading curve using the procedure proposed by Olivier and Pharr [25]. The distances between measurements were, successively, 0, 450, 900, 1350, and 1800 µm. In this article, the results of nanotesting the composite containing 0.5 wt.% TPO are not stated because when the samples were cured on one side, the bottom stayed uncured. The bottom of these samples remained a soft paste.

The diametral tensile strength test (DTS) assesses the tensile properties of the dental composite materials. Eleven cylindrical samples (diameter 6 mm and thickness 3 mm) were tested of every composite. DTS is the maximum resistance against loads tending to destroy a sample. The crosshead speed was 2 mm/min. The examination was determined with universal testing machine (Z020, Zwick/Röell, Ulm, Germany). The numerical value is calculated according to the following formula (1):(1)$\mathrm{DTS}\;[\mathrm{MPa}]=\frac{2P}{\pi DT}$
where:DTS—diametral tensile strength (MPa);P—load applied (N);D—diameter of sample (mm);T—high of sample (mm).

Flexural strength was performed on a three-point bending test. Six samples were tested of every composite. The samples used in this test were rectangular (25 mm × 2 mm × 2 mm), and they were irradiated at 3 points twice for 20 s on both sides: In total, the sample was irradiated for 120 s. The samples were then placed on two supports 20 mm apart. A force was applied in the middle, downwards at a 90° angle. The test was evaluated in a universal testing machine (Zwick Z020, Zwick/Röell, Ulm, Germany), the crosshead speed was 1 mm/min and the examination complied with ISO regulations [26]. The maximum force, which destroyed the sample, was measured for each specimen.

Flexural strength (MPa) was defined with following Equation (2):(2)$\mathrm{FS}\;[\mathrm{MPa}]=\frac{3Wl}{2bh^{2}}$
where

W—force, which caused the destruction of the sample (N);l—distance between supports, 20 mm;b—width of sample (mm);h—high of sample (mm).

The last examination which was performed was CIE L* a* b* color system on spectrophotometer KONICA MINOLTA CM-3600A (Germany). Every color can be described by three values: hue, brightness and saturation. This test was performed on cylindrical samples (2 mm height and 10 mm diameter) that were cured before testing. The three measurement were made for each composite. The spectrophotometer was calibrated in accordance with the manufacturers’ recommendation. The control group was sample containing CQ and DMAEMA as a photoinitiator system. System CIE L* a* b* contains three axes: a* and b*, which are at right angles to each other and define the basic colors. The third axis L* means brightness and it is perpendicular to plane created by axes a* and b*. A scale of axis a* is from −120 (green color) to +120 (red color). A scale of axis b* is from −120 (blue color) to +120 (yellow color). The scale of axis L* is from 0 (black saturation) to 100 (white saturation). System CIE L* a* b* assumes considering the differences between colors on basis of the distance between points in the three-axes spatial layout.

For the statistical analysis of the results, Microsoft Excel from the Microsoft Office 2010 and Statistica v. 13 (Statsoft Polska Sp.z o. o., Krakow, Poland) were used. To evaluate the distributions of particular parameters, the Shapiro–Wilk test of normality was applied. In cases of nonnormal distribution, the Kruskall–Wallis test was used. In case of normal distribution of particular parameters, the equality of variances was assessed with the use of Levene’s test. For equal variances, ANOVA with Scheffe’s post hoc test was applied. The accepted level of significance was *p* = 0.05.

## 3. Results

**Figure 1 ijms-23-11594-f001:**
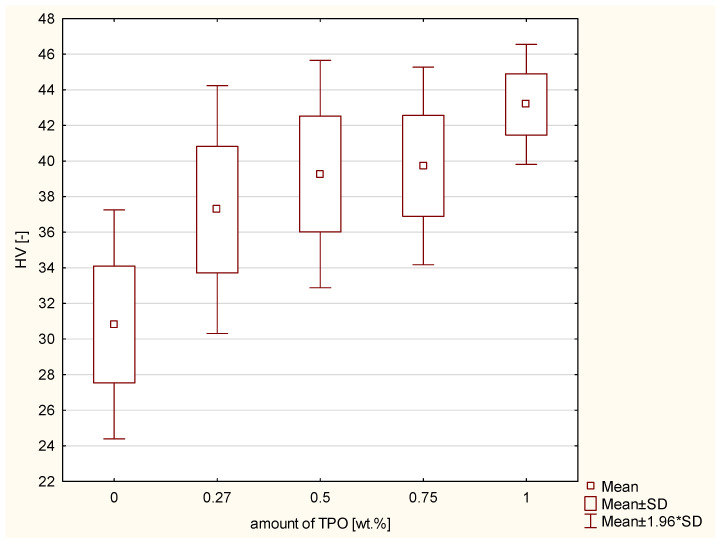
Box-and-whisker plot of Vicker’s hardness of composites containing CQ or TPO as photoinitiator; photopolymerization both side of samples 20 s per 2 mm thickness of composites with Valo lamp light intensity 1450 mW/cm^2^.

All results are summarized in Table A1, but individual studies (i.e., HV, DTS, etc.) are discussed separately for a more accurate analysis of the relationships. The first examination that was performed was Vicker’s hardness test. The control group containing CQ and DMAEMA had the lowest HV. The tendency was noticeable that the higher the concentration of TPO, the higher the HV. According to the ANOVA, a statistically significant difference was demonstrated in the HV (*p*-value = 0.00000) of manufactured composites. The post hoc Scheffe’s test showed statistically significant differences (Figure 1) between:Composite including CQ and 0.27 wt.% TPO (*p*-value = 0.00032), with higher values of experimental composite with 0.27 wt.% TPO.Composite including CQ and 0.5 wt.% TPO (*p*-value = 0.00000), with higher values of experimental composite with 0.5 wt.% TPO.Composite including CQ and 0.75 wt.% TPO (*p*-value = 0.00000), with higher values of experimental composite with 0.75 wt.% TPO.Composite including CQ and 1 wt.% TPO (*p*-value = 0.00000), with higher values of experimental composite with 1 wt.% TPO.Composite containing 0.27 and 1 wt.% of TPO (*p*-value = 0.00117), with higher values for material with 1 wt.% TPO.

**Figure 2 ijms-23-11594-f002:**
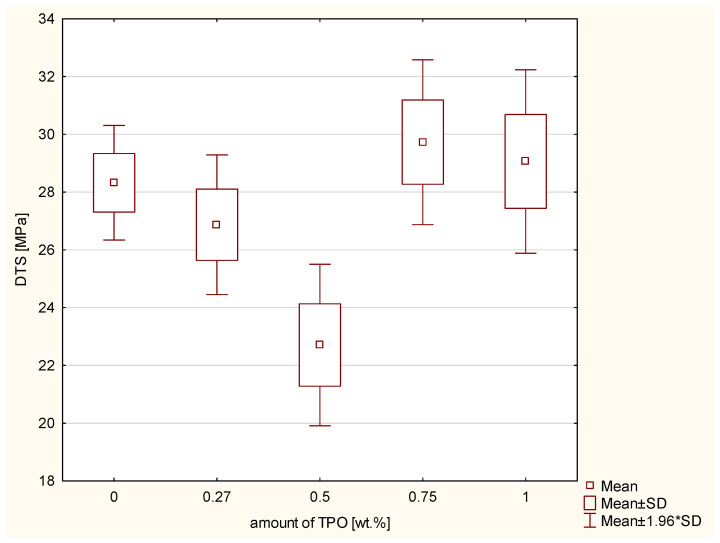
Box-and-whisker plot of diametral tensile strength (DTS) [MPa] of experimental composites including different concentration of TPO and values of control group–CQ and DMAEMA; photopolymerization both side of samples 20 s per 2 mm thickness of composites with Valo lamp light intensity 1450 mW/cm^2^.

The highest microhardness on the top of sample characterize the composite with 0.27 wt.% of TPO. The lowest microhardness on the top of sample characterize the composite with 1 wt.% of TPO. On the high 1800 μm the lowest microhardness and reduced modulus has composite with conventional photoinitiator—CQ. Inside the sample the highest values of microhardness has composite containing CQ (Table 2). The second test was diametral tensile strength. The values for the control group were not the lowest; rather, they were in the optimal middle of the chart. However, there is no tendency concerning dependence values of DTS from concentration of TPO. According to the ANOVA, a statistically significant difference was demonstrated in the DTS (*p*-value = 0.00521). The post hoc Scheffe’s test showed statistically significant differences (Figure 2) between:Composite containing TPO 0.5 wt.% and 0.75 wt.% of TPO (*p*-value = 0.01763), with higher values for composite with 0.75 wt.% TPO.Dental material including TPO 0.5 wt.% and 1 wt.% of TPO (*p*-value = 0.04058), with higher values of material with 1 wt.% TPO.

**Figure 3 ijms-23-11594-f003:**
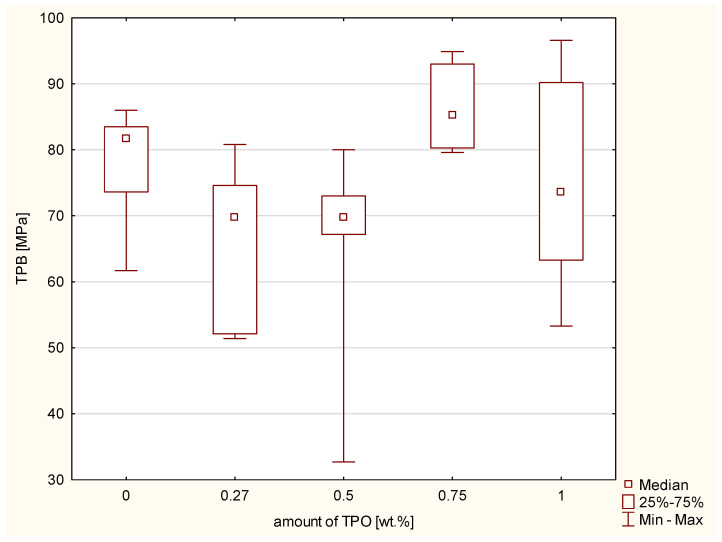
Box-and-whisker plot of three-point bending flexural strength (FS) of experimental composites containing different concentration of TPO and the control group CQ and DMAEMA; photopolymerization both side of samples 20 s per 2 mm thickness of composites with Valo lamp light intensity 1450 mW/cm^2^.

**Figure 4 ijms-23-11594-f004:**
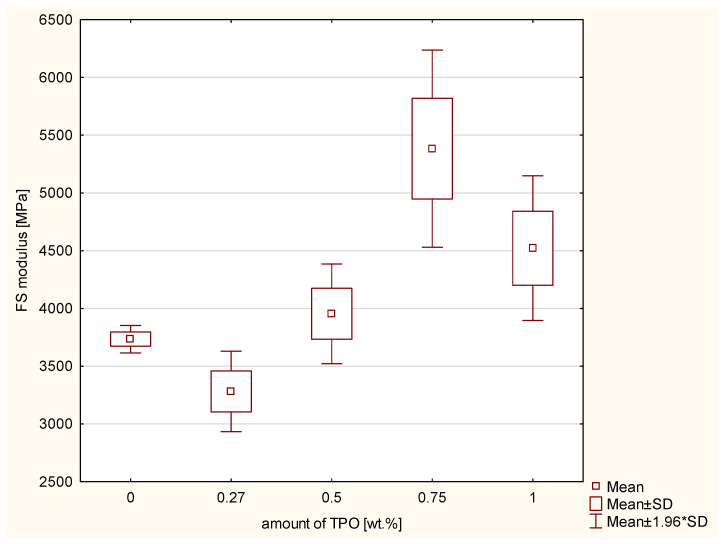
Box-and-whisker plot of s of the modulus of elasticity in bending (FS modulus) [MPa] between composites with different photoinitiator systems; photopolymerization both side of samples 20 s per 2 mm thickness of composites with Valo lamp light intensity 1450 mW/cm^2^.

Next the three-point bending test was conducted, which determines the flexural strength and the modulus of elasticity in bending. According to the Kruskal–Wallis test, a statistically significant difference was not demonstrated in the TPB (*p*-value = 0.05000). The results are shown in Figure 3. However, according to the ANOVA, there was a statistically significant difference in the modulus of elasticity in bending (FS modulus) (*p*-value = 0.00023). The increasing values of flexural strength modulus are connected with higher concentration of TPO. The post hoc Scheffe’s test showed statistically significant differences (Figure 4) between the FS modulus of:Composite including CQ and 0.75 wt.% TPO (*p*-value = 0.01319), with higher values of experimental composite with 0.75 wt.% TPO.Material with 0.27 wt.% and 0.75 wt.% TPO (*p*-value = 0.00065), with higher values of material with 0.75 wt.% TPO.Composites including TPO 0.5% and TPO 0.75% (*p*-value = 0.02816), with higher values in TPO 0.75%.

**Figure 5 ijms-23-11594-f005:**
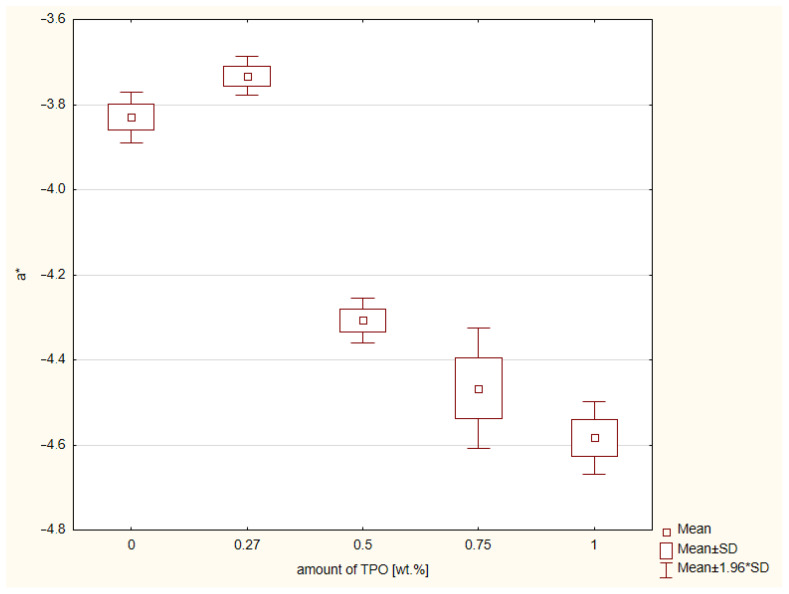
Box-and-whisker plot of results of color measurement CIE L* a* b* according to the axis a* of experimental composites containing different concentration of TPO and the control group CQ and DMAEMA; photopolymerization both side of samples 20 s. per 2 mm thickness of composites with Valo lamp light intensity 1450 mW/cm^2^.

**Figure 6 ijms-23-11594-f006:**
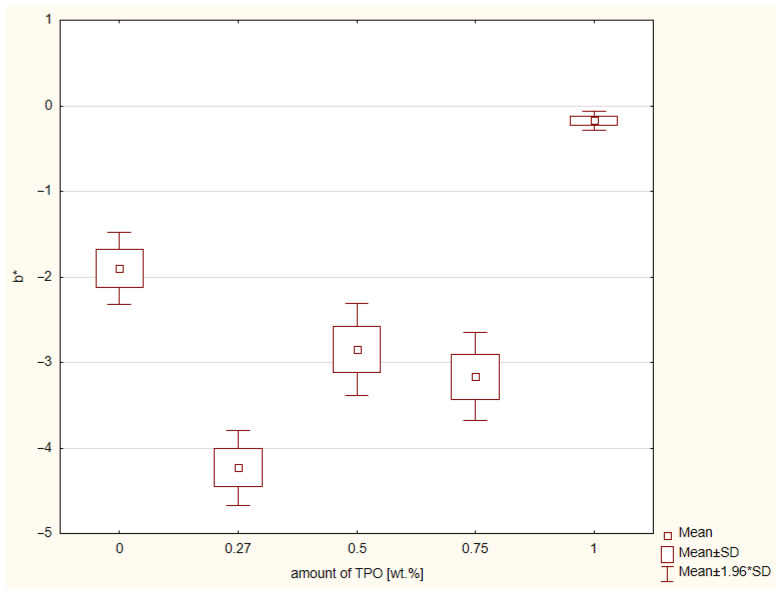
Box-and-whisker plot of results of color measurement CIE L* a* b* according to the axis b* of experimental composites containing different concentration of TPO and the control group CQ and DMAEMA; photopolymerization both side of samples 20 s. per 2 mm thickness of composites with Valo lamp light intensity 1450 mW/cm^2^.

The results of CIE L* a* b* color system are shown in Figure 5 and Figure 6. There were three color measurements made for each composite. The values closest to the control group of CQ and DMAEMA on axis a* are connected with concentration of TPO. The higher the TPO concentration, the greater the distance between control group and research group. According to the ANOVA, a statistically significant difference was demonstrated in the a* (*p*-value = 0.00000). The post hoc Scheffe’s test showed statistically significant differences between:Composite with CQ and 0.5 wt.% of TPO (*p*-value = 0.00015), with higher values of material including CQ,Materials containing CQ and 0.75 wt.% TPO (*p*-value = 0.00001), with higher values of composite with CQ,Dental resin composite including CQ and 1 wt.% TPO (*p*-value = 0.00000), with higher values of material with CQ,Composite containing 0.27 wt.% and 0.5 wt.% TPO (*p*-value = 0.00001), with higher values of material with 0.27 wt.% of TPO,Resins with 0.27 wt.% and 0.75 wt.% of TPO (*p*-value = 0.00000), with higher values of composite including 0.27 wt.% of TPO,Dental materials with 0.27 wt.% and 1 wt.% of TPO (*p*-value = 0.00000), with higher values of material with 0.27 wt.% of TPO,Composites with 0.5 wt.% and 1 wt. % (*p*-value = 0.001207), with higher values of resin containing 0.5 wt.% of TPO.

There was no specific tendency between distance in the control group and the concentrations in the research groups. According to the ANOVA, a statistically significant difference was demonstrated in b* (*p*-value = 0.00000). The post hoc Scheffe’s test showed statistically significant differences (Figure 6) between:Composite containing CQ and 0.27 wt.% TPO (*p*-value = 0.00022), with higher values for resin with CQ.Dental resin including CQ and 0.75 wt. %TPO (*p*-value = 0.03649), with higher values for material with CQ.Material with CQ and 1 wt.% TPO (*p*-value = 0.00472), with higher values for composite with 1 wt.% of TPO.Dental composite including 0.27 wt.% and 0.5 wt.% TPO (*p*-value = 0.01404), with higher values for resin with 0.5 wt.% of TPO.Resin based composite with 0.27 wt.% and 1 wt.% TPO (*p*-value = 0.00000), with higher values for composite 1 wt.% of TPO.Material containing 0.5 wt.% and 1 wt.% TPO (*p*-value = 0.00012), with higher values for resin based composite with 1 wt.% of TPO.Composite with 0.75 wt.% and 1 wt.% TPO (*p*-value = 0.00004), with higher values for material containing 1 wt.% of TPO.

## 4. Discussion

This article compares the various features of dental composites containing CQ/DMAEMA and different concentration of TPO. This range of analyses highlights the most important properties of dental composites. The null hypothesis of our research was accepted that dental resin containing TPO performs no worse than composites with CQ/tertiary amines.

The first examination that was performed was Vicker’s hardness. It was statistically confirmed that composites with TPO as a photoionitiator show higher values of hardness. Moreover, the higher concentration of TPO, the better the values of hardness. This tendency is also confirmed in other research. Salgado et al. [23] performed the Knoop analysis of hardness and proved that dental resin containing CQ (1.0 mol%) and ethyl 4-dimethylaminobenzoate (1.0 mol%) showed lower hardness values than dental composite including TPO (1.0 mol%). Randolph et al. [14] also confirmed that composites containing TPO (0.42 wt.%) has higher values of hardness than CQ with dimethylaminoethylmethacrylate (0.2/0.8 wt.% appropriately) regardless of the exposure time. Frequently, hardness is used as an indirect measurement of degree of conversion and as an indicator of cross-linking [27]. Bertolo et al. showed that composite containing TPO (0.5 mol%) had a higher degree of conversion than did material with CQ and EDMAB (0.5 mol%/1 mol%). For TPO, it was 63.0 ± 1.0% and for CQ/EDMAB, 56.05 ± 0.5% [27]. Our findings are in line with above mentioned results. Randolph et al. also proved that TPO (0.42 wt.%) has a higher degree of conversion than CQ and DMAEMA (0.2/0.8 wt.%). Moreover, TPO achieves better results of degree of conversion in a shorter exposure time [21].

The test of nanoindentation shows that composite containing CQ/DMAEMA has the best values of hardness inside the samples, and the deeper the penetration within the sample, the better hardness values. However, the dental resin containing 0.27 wt.% of TPO manifests higher values of hardness than CQ/DMAEMA at distance 450 µm and it retains a similar growth tendency as CQ/DMAEMA. Inside the samples, at 900 µm and 1350 µm, the highest hardness value other than that of CQ/DMAEMA was TPO 0.75 wt.%. According to Salgado et al., research composites including TPO has lower depth of cure values than dental resin with CQ and DMAEMA system [23]. Palin et al. tested commercial composites containing different photoionitiator systems with addition of TPO such as TetricCeram, Tetric EvoCeram and Tetric Bleach. They also proved that composite containing only CQ (Tetric) and tertiary amine has better values of hardness inside the samples and this conclusion were confirmed regardless of the dental lamp used. Comparing the results of Vicker’s hardness when samples were cured with LED lamp: Tetric average value is top 78.5 (±3.1) and bottom 76.7 (±3.4), TetricCeram top 68.7 (±3.2) and bottom 60.9 (±3.4), Tetric EvoCeram top 56.8 (±2.0) and bottom 53.4 (±1.9) and Tetric Bleach top 43.2 (±2.1) and bottom 37.8 (±2.8) [28]. The similar research was performed by Par et al. [29]. They tested Knoop hardness of commercial composites containing different photoinitiator system comparing to Filitek One Bulk Fill. The samples were cured in different protocols but the results of hardness were higher for composites containing CQ. The results of Knoop hardness of Filtek One Bulk Fill crossed 60 value of Knoop’s hardness, although Tetric EvoCeram Bulkfill and Tetric Powerfill do not reach 60 value of Knoop’s. This might be caused by the different protocol of cure, than in our research [29]. The same conclusion was proved also in our last research [8]. Filtek Ultimate containing golden mean–CQ and tertiary amine has higher values of microhardness inside the sample (1570 ± 160 MPa) than Tetric EvoCeram Powerfill (1260 ± 200 MPa) and Tetric EvoCeram Bleach (1250 ± 170 MPa) which include not only CQ, but also TPO and Ivocerin [8]. Ivocerin —dibenzoyl germanium is a patented photoinitiator of one manufacturer Vivadent and it can be found only in their composites. The absorption range of Ivocerin is 390–445 nm and absorbance maximum is 418 nm. Ivocerin forms at least two radicals, that means it is also more efficient than CQ [8,22,30]. Our research due to nanoindentation shows that composites with TPO have lower values of hardness inside samples than those with CQ. However, the highest concentration of TPO, the highest values of hardness inside the samples. But it is worth underlining again that composite containing 0.5 wt.% TPO as a photoinitiator was not cured on unexposed side, when the sample was 2 mm high. The depth of cure depends on the concentration of photoinitiator.

The nanoindentation test not only assesses the microhardness but also reduced modulus of elasticity. Inside sample containing CQ/DMAEMA reduced modulus is growing with the distance of measurement. This trend is the same as microhardness.

The diametral tensile strength test shows that resins containing higher values than 0.5 wt.% of TPO manifests higher values of DTS than composites with CQ/DMAEMA. However, composite with 0.27 wt.% TPO shows not much lower value of DTS than CQ/DMAEMA. Delgado et al. in their studies were testing cements containing different photoinitiator systems: TPO in concentration 0.4 wt.% and CQ with amine in concentration 0.2 wt.% and 0.2 wt.% accordingly. They assessed the ultimate tensile strength of the cements which were tested in two layers (0.7 mm and 1.5 mm). The cement containing CQ and amine has higher values of tensile strength than TPO. The mean results for layer 0.7 mm for CQ was 28.296 ± 2.54 MPa and for TPO was 22.356 ± 2.42 MPa. For layer 1.5 mm for CQ was 21.566 ± 2.70 MPa and for TPO 14.896 ± 3.60 MPa [31]. Considering the results of diametral tensile strength of TPO 0.27 wt.% and 0.5 wt.% and comparing them to results of CQ/DMAMEA, the numerical results of golden means are better than TPO in this low concentration. This dependence seems convergent with presented study.

The results of three-point bending strength test did not show significant statistical differences between all composites. However, the highest values of flexural strength has composite containing 0.75 wt.% of TPO. This composite has also the highest values of FS modulus. Randolph et al. shows also that dental resin containing TPO (0.42 wt.%) has higher flexural modulus than material with CQ with dimethylaminoethylmethacrylate (0.2/0.8 wt.%) regardless of the time exposure and the power of dental curing unit [21]. Delgado et al. testes also flexural strength of cements containing TPO in concentration 0.4 wt.% and CQ with amine in concentration 0.2 wt.% and 0.2 wt.%. Despite the thickness of layer, the CQ has better flexural strength than 0.4 wt.% TPO. The results for 0.7 mm layer for CQ were 105.256 ± 8.97 MPa, for TPO 93.816 ± 5.81 MPa and for 1.5 mm layer were 88.946 ± 4.84 and 69.72 ± 5.81 MPa [31]. Considering the results of flexural strength of TPO 0.27 wt.% and 0.5 wt.% and comparing them with the results for CQ/DMAMEA, the results of golden mean are better than TPO in this low concentration.

The last examination was CIE L* a* b*. This method is indicated for determination the shade of dental materials, not only composites but also ceramic and let scientists compare these material shades with color of dentine or enamel of natural teeth. The axis a* refers to color green and red. There was also significant statistical difference in values of a*. The CQ/DMAEMA is a control group and reference point. The color of composite with CQ/DMAEMA is closer to red, so this hue is warmer than the hue of composites containing TPO in concentrations 0.5 wt.%, 0.75 wt.%, and 1 wt.%. However, the composite containing 0.27 wt.% TPO has hue values deviated to green color. The closest values to red had composite including 1 wt. % of TPO. Axis b* refers to yellow and blue. The composite containing CQ/DMAEMA has color values closer to yellow than composites containing TPO in concentration 0.27 wt.%, 0.5 wt.% and 0.75 wt.%. The hue of composite including 0.27 wt.% TPO were the most distant from yellow. In contrast, composite containing 1% TPO had the closest values to yellow, even closer than CQ/DMAEMA. The lower the concentration of TPO, the less yellow the composite. Bertolo et al. [24] also analyzed the color of the composites containing alternative photoinitiator systems. The concentration of TPO was 0.5 mol% and 0.5 mol%/1.0 mol% of CQ and ethyl-4-(dimethylamino)benzoate (EDMAB). The highest yellow values according to their studies has composites containing CQ/EDMAB, so this statement also confirms our results. Not only do Bertolo et al.’s results confirm our conclusions, Salgado et al. [23] also showed that composite containing TPO (1.0 mol%) was less yellow than composite with CQ/EDMAB (1.0 mol%/1.0 mol%). In clinical use, this less yellowish composite will match with ultra-white teeth after bleaching.

## 5. Conclusions

All the composites were tested to assess the most important mechanical properties, and the hues of the composites were also rated. The concentration of photoinitiator influenced the Vicker’s hardness value. Additionally, samples containing TPO had better hardness values. There was also a noticeable influence of type and concentration of photoinitiator on the diametral tensile strength. To sum up: the best mechanical and aesthetic properties were found in the composite containing 0.75 wt.% TPO as a photoinitiator. This composite had higher microhardness, diametral tensile strength, flexural strengths and modulus of elasticity than the composite with the conventional/often used photoinitiator system, i.e., CQ with tertiary amine. Not only were the mechanical properties better, but this composite was less yellow than material with CQ/DMAEMA. However, to answer the question in the title, more tests on the composite with TPO need to be done, i.e., conversion degree measurements, shrinkage stress analysis and material cytotoxicity tests.

## References

## Figures and Tables

**Table 1 ijms-23-11594-t001:** The composition of the experimental composites tested in this study-55 wt.% of matrix involving Bis-GMA and TEGDMA (60/40 ratio) and 45 wt.% of silanized silica filler.

Group	Photoinitiator System	Manufacturer	Concentration of Photoinitiator
A.	CQ and DMAEMA	Sigma-Aldrich Inc., St. Louis, MO, USA	0.4 wt.% and 0.8 wt.%
B.	TPO		0.27 wt.%
C.	TPO		0.5 wt.%
D.	TPO		0.75 wt.%
E.	TPO		1 wt.%

**Table 2 ijms-23-11594-t002:** The average results for microhardness (MPa) and reduced modulus (GPa) with standard deviations. The results were determined by nanoindentation of the top (0 μm), bottom (1800 μm), and cross-section (450, 900 and 1350 μm) of tested material samples; photopolymerization one side 20 s per 2 mm with Valo lamp light intensity 1450 mW/cm^2^.

Distance [µm]	CQ/DMAEMA		TPO 0.27 wt.%		TPO 0.75 wt.%		TPO 1 wt.%	
	Microhardness [MPa]	Reduced Modulus [GPa]	Microhardness [MPa]	Reduced Modulus [GPa]	Microhardness [MPa]	Reduced Modulus [GPa]	Microhardness [MPa]	Reduced Modulus [GPa]
0	67.52 ± 2.5	2.15 ± 0.1	109.58 ± 2.6	0.53 ± 0.1	57.58 ± 2.5	0.3 ± 0.0	44.97 ± 5.8	0.69 ± 0.0
450	352.5 ± 55.4	5.38 ± 0.4	372.77 ± 34.7	4.08 ± 0.3	108.66 ± 10.5	1.8 ± 0.1	336.67 ± 50.8	4.08 ± 0.5
900	671.43 ± 89.9	5.51 ± 0.1	486.34 ± 9.3	5.07 ± 0.1	590.06 ± 3.6	6.9 ± 0.9	423.08 ± 10.1	3.93 ± 0.5
1350	753.49 ± 2.9	8.34 ± 0.6	478.84 ± 69.2	5.00 ± 0.5	499.52 ± 43.1	5.5 ± 0.3	280.53 ± 4.3	3.21 ± 0.2
1800	12.2 ± 1.5	0.72 ± 0.0	50.25 ± 1.1	2.08 ± 0.3	255.98 ± 3.8	4.00 ± 0.4	210.4 ± 5.9	3.12 ± 0.4

## Data Availability

The data presented in this study are available on request from the corresponding author.

## Appendix A

**Table A1 ijms-23-11594-t0A1:** Median values of Vicker’s hardness, diametral tensile strength (DTS), three-point bending flexural strength (TPB), and modulus of elasticity in bending (FS modulus) with standard deviation for tested composites.

Composite	Vicker’s Hardness	Standard Deviation	DTS [MPa]	Standard Deviation	TPB [MPa]	Standard Deviation	FS Modulus [MPa]	Standard Deviation
CQ + DMAEMA	30.82	3.3	28.32	3.4	77.3	9.9	3734.00	136.7
TPO 0.27 wt.%	37.27	3.6	26.87	4.1	66.38	12.4	3281.67	435.4
TPO 0.5 wt.%	39.27	3.3	22.7	4.7	65.4	16.6	3953.33	540.0
TPO 0.75 wt.%	39.73	2.8	29.73	4.8	86.4	6.6	5383.33	1067.1
TPO 1 wt.%	43.28	1.7	29.06	5.4	75.1	16.2	4521.67	783.4
